# Spectral Fingerprinting of Tencha Processing: Optimising the Detection of Total Free Amino Acid Content in Processing Lines by Hyperspectral Analysis

**DOI:** 10.3390/foods13233862

**Published:** 2024-11-29

**Authors:** Qinghai He, Yihang Guo, Xiaoli Li, Yong He, Zhi Lin, Hui Zeng

**Affiliations:** 1 School of Mechanical Engineering, Qilu University of Technology (Shandong Academy of Sciences), Jinan 250353, Chinaguoyihang_gyh@163.com (Y.G.); 2 Shandong Academy of Agricultural Machinery Science, Jinan 250100, China; 3 College of Biosystems Engineering and Food Science, Zhejiang University, Hangzhou 310058, China; 4 Tea Research Institute, Chinese Academy of Agricultural Sciences, Hangzhou 310008, China; 5 Hangzhou Jingle Tea Foundation, Hangzhou 310000, China; zhong13575477242@126.com

**Keywords:** hyperspectral imaging technology, Tencha, spreading, fixing, drying, total free amino acid content

## Abstract

The quality and flavor of tea leaves are significantly influenced by chemical composition, with the content of free amino acids serving as a key indicator for assessing the quality of Tencha. Accurately and quickly measuring free amino acids during tea processing is crucial for monitoring and optimizing production processes. However, traditional chemical analysis methods are often time-consuming and costly, limiting their application in real-time quality control. Hyperspectral imaging (HSI) has shown significant effectiveness as a component detection tool in various agricultural applications. This study employs VNIR-HSI combined with machine learning algorithms to develop a model for visualizing the total free amino acid content in Tencha samples that have undergone different processing steps on the production line. Four pretreating methods were employed to preprocess the spectra, and partial least squares regression (PLSR) and least squares support vector machine regression (LS–SVR) models were established from the perspectives of individual processes and the entire process. Combining competitive adaptive reweighted sampling (CARS) and variable iterative space shrinkage approach (VISSA) methods for characteristic band selection, specific bands were chosen to predict the amino acid content. By comparing modeling evaluation indicators for each model, the optimal model was identified: the overall model CT+CARS+PLSR, with predictive indicators Rc^2^ = 0.9885, Rp^2^ = 0.9566, RMSEC = 0.0956, RMSEP = 0.1749, RPD = 4.8021, enabling the visualization of total free amino acid content in processed Tencha leaves. Here, we establish a benchmark for machine learning-based HSI, integrating this technology into the tea processing workflow to provide a real-time decision support tool for quality control, offering a novel method for the rapid and accurate prediction of free amino acids during tea processing. This achievement not only provides a scientific basis for the tea processing sector but also opens new avenues for the application of hyperspectral imaging technology in food science.

## 1. Introduction

Tencha originated in the Sui Dynasty of China and is typically used as the initial raw material for matcha processing. Total Free Amino Acid in Tencha, especially glutamic acid and theanine (γ-glutamylethyl-amide), not only enhance the freshness and sweetness of tea taste [1] but also have a calming effect on nerves and anti-fatigue properties [2]. With the pursuit of health and unique matcha beverages, Tencha has gained global attention once again.

Compared to traditional brewed tea, Tencha, which can be consumed both as a drink and as a food ingredient, places greater emphasis on the aroma and color of the tea rather than its shape and luster. Consequently, its processing methods differ slightly. Before processing, tea plants used for Tencha undergo strict shading treatment for 7 to 21 days prior to harvest. This treatment suppresses catechin synthesis and increases the levels of free amino acids and chlorophyll in the leaves [3], thereby enhancing the vibrant green color and sweet taste of Tencha [4] and imparting its distinctive “seaweed-like aroma” [5,6]. Additionally, Tencha requires multiple processing steps, including fresh leaf spreading, steaming, drying, and separating stems and leaves. Among these, steaming and drying are crucial steps in the processing process. Steaming quickly deactivates the enzymes in fresh leaves, preserving their color, while drying reduces moisture content, making the tea easier to store and transport, and also intensifies its unique flavor and aroma. Therefore, studying changes in amino acid content during processing is vital for improving the quality of Tencha.

Traditionally, the conventional detection methods for the internal components of tea include High Performance Liquid Chromatography (HPLC) [7], spectrophotometry, Gas Chromatography–Mass Spectrometry (GC–MS) [8], and Capillary Electrophoresis (CE) [9,10]. These methods offer high precision and sensitivity [11], but they are cumbersome, time-consuming, expensive, and often disrupt sample tissues, making real-time analysis data acquisition impractical [12]. Therefore, there is an urgent need to develop a new method that can conduct rapid routine analysis and achieve real-time monitoring of industrial quality to meet the fast detection requirements of Tencha production and processing.

VNIR (Visible and Near-Infrared) Hyperspectral imaging technology has been widely applied in the field of crops. It combines the intuitiveness of traditional imaging techniques with the precise analytical capabilities of spectroscopy, providing detailed spatial distribution and spectral information for the object being tested simultaneously [13,14]. Therefore, as a rapid and non-destructive analytical method, VNIR–HSI technology has attracted widespread attention in tea detection research. In tea classification studies, researchers can accurately identify tea varieties [15], assess storage time [16], identify adulterants [17], and determine tea quality grades [18]. In terms of quantitative analysis, this technology has made significant achievements in predicting the content of catechins [19], various monomers [20], tea polyphenols, caffeine [21], and other key components in tea. However, most current research focuses on the detection of traditional finished teas or commercial teas, with relatively little research on semi-finished teas during the processing stage.

In this study, a prediction method for Total Free Amino Acid (TFAA) in Tencha based on VNIR–HSI under different processing steps is proposed. First, a hyperspectral acquisition system was established to obtain hyperspectral images of Tencha samples, and the corresponding physicochemical values of free amino acids were determined. Then, spectral information was extracted from these images and subjected to various pretreating methods for initial treatment. Subsequently, competitive adaptive reweighted sampling (CARS) and variable iterative space shrinkage approach (VISSA) were employed to extract characteristic wavelengths. Finally, optimal prediction models for TFAA in Tencha were constructed using partial least squares regression (PLSR) and least squares support vector machine regression (LS-SVR), based on both detailed models of each processing step and an overall model across all steps.

## 2. Materials and Methods

### 2.1. Tea Sample Collection

**Figure 1:**
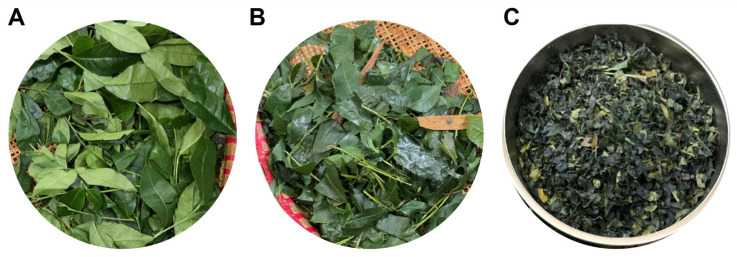
Three processing steps of Tencha: (**A**) fresh leaves spreading, (**B**) steaming fixation, and (**C**) hot air drying.

The selected tea variety for this study was Jiukeng (JK) from Hangzhou City, Zhejiang Province, as shown in Figure 1. The processing steps for JK include fresh leaves spreading (FLS), steaming fixation (SF), and hot air drying (HD). Specifically, freshly picked leaves were first naturally spread in a tea withering trolley. The process parameters maintained in the equipment were a temperature of 18 °C, relative humidity of 60%, and a leaf thickness of 2–4 cm. Mixing was conducted every 30 min to ensure uniform heat conduction within the leaves during processing. Subsequently, the withered leaves were fed into a 300K-SS type rolling machine (Kawasaki Heavy Industries, Ltd., Kobe, Japan), adjusting the steam flow rate to approximately 145 kg/h based on the temperature and humidity conditions of the workshop that day. The inclinations of the tender and old leaves in the steam drum were set at 5–8° and 2–4°, respectively, with stirring shaft speeds of approximately 400 rpm and drum speeds of about 40 rpm.

Finally, the processed leaves were placed into a tea dryer for multiple drying cycles, concluding all processing steps. Meanwhile, it is important to note that in the FLS step, as shown in Figure 1A, the samples were prepared by evenly spreading freshly harvested Tencha leaves for natural air exposure. This process allows for a slight reduction in leaf moisture and mild transformation of internal compounds, maintaining the fresh green color of the leaves. After the completion of all processing steps, experimental sampling was carried out. In total, 270 sets of JK samples were obtained, comprising 3 (processing techniques) × 30 (sampling batches) × 3 (replicate sampling times).

### 2.2. Spectral Collection

**Figure 2:**
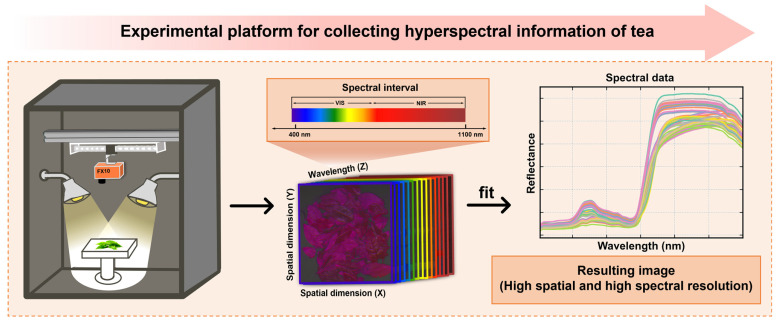
Experimental platform for collecting hyperspectral information.

A homemade dark box spectroscopy acquisition experimental platform (Figure 2) was used, equipped with a line-scan hyperspectral camera Specim FX10 (Specim, Spectral Imaging Ltd., Oulu, Finland) with a wavelength range of 400 nm to 1000 nm, spectral accuracy of ±1 nm, and a resolution of 4 cm^−1^. This setup was employed to capture spectral images of tea samples after undergoing three processes (FLS, SF, and HD). The Tencha samples used in this study were those prepared and described in 2.1, with each sample consisting of an equal mass of leaves. The samples were evenly spread on an immovable platform to ensure consistent imaging conditions, and one hyperspectral image was captured for each sample using the spectroscopy camera, which moved linearly at a constant speed via a screw mechanism.

To ensure the stability and accuracy of spectral images, critical parameters of the experimental platform, such as camera movement speed, exposure time, and focal length, were adjusted. To eliminate the influence of uneven light distribution and dark current on hyperspectral images, dark current information was first collected before capturing the spectral images of the samples, together with a standard barium sulfate whiteboard. After each sample was collected, it was placed into a high-throughput rapid grinding machine (JXFSTPRP-48L, Jingxin, Shanghai) for centrifugal grinding under identical conditions. Finally, powdered samples were filtered through a 60-mesh sieve, sealed in envelopes, and stored frozen.

### 2.3. Chemical Analysis

According to the Chinese National Standard (GB/T 8314-2013) [22], the amino acid chemical indices in the samples were determined using the ninhydrin colorimetric method. Firstly, 0.25 g of sample was weighed into a 2.0 mL centrifuge tube, to which 1.0 mL of distilled water was added, followed by a 5-min water bath at 100 °C, and subsequent cooling and centrifugation. A measurement of 1.0 mL of the supernatant was taken, to which 0.5 mL of 2% ninhydrin solution and 0.5 mL of pH 8.0 phosphate buffer were added, and absorbance A at 570 nm was measured. Simultaneously, tea amino acid was prepared as a control group for each experiment, and a standard curve was plotted for comparative evaluation of the TFAA content in the samples.

(1) $$TFAA=\frac{10^{-3}\times C_{0}\times V_{1}}{M\times m\times V_{2}}\times 100\%$$

The final amino acid content was expressed as a dry mass fraction and was calculated with reference to the Formula (1), where *TFAA* is the content of total free amino acid, *V*_1_ is the total amount of the test solution (mL), *V*_2_ is the total amount of the specimen used for the determination (mL), *M* is the mass of the specimen (g), *C*_0_ is the mass of teicoplanin obtained from the standard curve on the basis of the measured absorbance A, and *m* is the percentage of dry matter content of the specimen (%).

### 2.4. Spectral Information Extraction

#### 2.4.1. Average Reflectance Spectrum Extraction and Pre-Processing

After collecting hyperspectral images, in order to reduce the influence of light sources and dark currents on the spectral information of samples, it is necessary to use the reflectance data of extracted samples, whiteboard, and dark current. By applying a formula, the average spectrum of tea samples (i.e., average reflectance) is corrected. Here, R_S_ represents the corrected spectrum of the sample, R_raw_ represents the raw spectrum, and R_w_ and R_b,_ respectively, denote the average spectra of whiteboard and dark current.

(2) $$R_{s}=\frac{R_{\mathrm{raw}}-R_{b}}{R_{w}-R_{b}}$$

Prior to determining characteristic wavelengths, in order to improve the spectral characteristics of the original spectrum and effectively eliminate baseline drift and noise caused by interference factors during the collection process, we employed various pretreating methods. These methods include multi-source scattering correction (MSC), mean centering (CT), first-order derivative (D1), and Savitzky–Golay smoothing (SG).

#### 2.4.2. Selection of Characteristic Wavelengths

Original hyperspectral images typically exhibit high-dimensional and multi-band characteristics, which may include redundant bands irrelevant to target information. Prior to spectral modeling, selecting characteristic bands can reduce data redundancy and overfitting risks, while enhancing model robustness and generalization capability. Therefore, this study employs two characteristic wavelength selection strategies, Competitive Adaptive Reweighted Sampling (CARS) and Variable Iterative Space Shrinkage Approach (VISSA), for comparative data processing.

The CARS algorithm effectively reduces the high collinearity among spectral bands by selecting characteristic variables, thereby improving the accuracy and speed of prediction models [23]. This algorithm treats each band variable as an individual entity and retains those with strong adaptive capability during the selection process [24].

VISSA, as a novel screening method, demonstrates good predictive ability for data within the near-infrared wavelength range [25]. It utilizes weighted binary matrix sampling to generate sub-models spanning variable subspaces, optimizing the performance evaluation of variable spaces [26]. During the optimization process, the VISSA method emphasizes two key rules: first, gradually narrowing the variable space in each step; second, ensuring that the new variable space is superior to the previous one, distinguishing VISSA significantly from CARS. Ultimately, the combination of variables with the smallest root mean square error in the iterative results is selected as the screening outcome.

### 2.5. Building Calibration Models

#### 2.5.1. Modelling Data Segmentation

To accurately explore the best predictive model for TFAA after different processes, before employing various spectral pretreating, characteristic band selection, and modeling algorithm selection, a comprehensive process model (CP model) and three detailed process models (FLS model, ST model, and HD model) were established. Samples for the comprehensive process model were selected in the same proportion from each process sample, with the total number of samples matching those in the three detailed models of processes.

When establishing the four different models, the SPXY (sample set partitioning based on joint X-Y distances) algorithm was used to divide the dataset into training and testing sets in a 3:1 ratio. The SPXY algorithm is an improved sample data classification method based on the Kennard–Stone method, effectively covering multidimensional vector space. Relevant experiments have shown that this algorithm can capture data distribution and correlations, thereby enhancing the stability and predictive ability of the established near-infrared spectral models [27,28].

#### 2.5.2. Selection of Modelling Methods

In order to more accurately predict the TFAA content of Tencha under different processing steps, this study selected two modeling methods: Partial Least Squares Regression (PLSR) and Least Squares Support Vector Machine Regression (LS–SVR) which simultaneously possess linear and nonlinear properties.

PLSR, as one of the classic multivariate regression algorithms [29], is widely used to reduce the dimensionality of a large number of potentially correlated variables to mitigate multicollinearity issues [30]. It achieves this by transforming the original independent variables into a new set of latent variables and selecting the optimal principal components for regression analysis, cleverly integrating strategies of variable decomposition and maximization of correlation [31]. This method effectively establishes a linear relationship between spectral data (response variables) and TFAA content (predictor variables) by explaining the directions of maximum variance in the spectral response matrix space. Meanwhile, this study employed k-fold cross-validation (k = 10) to estimate the optimal number of principal components (nLVs) in the PLSR model. The dataset was divided into 10 equally sized folds, with each fold used once as the validation set while the remaining 9 folds served as the training set. This method provides a more stable and robust estimate of model performance compared to Leave-One-Out Cross Validation (LOO–CV) [32].

LS–SVR, as an improved form of Support Vector Regression (SVR), avoids the quadratic programming problem in SVR [33] and reduces the computational complexity of the model [34]. Through appropriate kernel function mapping, it transforms complex low-dimensional nonlinear regression problems into linear regression in high-dimensional space. Relevant studies have shown its robustness and stability in near-infrared spectral prediction models. This study selected the Gaussian kernel function (RBF) with strong nonlinear mapping capability, combined with ten-fold cross-validation and grid search (GS) hyperparameter optimization algorithm [35,36], to select and adjust kernel function parameters (gamma) and regularization parameters (C), thereby providing more accurate and reliable regression predictions.

#### 2.5.3. Evaluation INDICATORS for Modelling

In order to assess the accuracy of the established models and ensure consistency in model evaluation metrics, this experiment used the coefficient of determination (*R*^2^), root mean square error (*RMSE*), and relative percent deviation (*RPD*) to measure the quality of each prediction model [37]. These indicators are calculated by the following formula:

(3) $$R^{2}=1-\frac{\sum_{i}{\left({\hat{y}}_{i}-y_{i}\right)}^{2}}{\sum_{i}{\left(y_{i}-\bar{y}\right)}^{2}}$$

(4) $$\mathrm{RMSE}=\sqrt{\frac{1}{N}\sum_{i=1}^{N}{\left(y_{i}-{\hat{y}}_{i}\right)}^{2}}$$

(5) $$RPD=\sqrt{\frac{\sum_{i-1}^{n}{\left({\hat{y}}_{i}-\bar{y}\right)}^{2}}{\sum_{i-1}^{n}{\left({\hat{y}}_{i}-{\bar{y}}^{*}\right)}^{2}}}$$

where $y_{i}$ and ${\hat{y}}_{i}$ represent the actual and model-predicted content of TFAA, respectively, while $\bar{y}$ and ${\bar{y}}^{*}\;$ represent the overall means of the actual and model-predicted content of TFAA, respectively. Meanwhile, N denotes the sample size. Specifically, an excellent prediction model should have a high *R*^2^ value close to 1 and a low RMSE value close to 0 in both the prediction and validation sets. Additionally, a larger RPD value indicates a more reliable model. If the RPD value falls between 1.4 and 2.0, the model is considered credible; if the RPD value exceeds 2.0, it indicates high credibility of the model and its suitability for further modeling analysis.

## 3. Results and Discussion

### 3.1. Spectral Characterization and Analysis of TFAA Content

**Figure 3:**
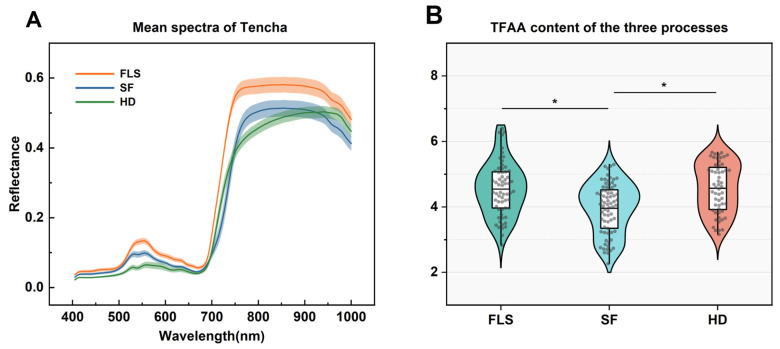
The mean spectra of different processes for Tencha (**A**); amino acid content of JK samples after different processes (**B**).

This study initially gathered raw spectral data through an acquisition and calibration process covering a wavelength range from 400 to 1000 nm. The spectral data consisted of 951 data points, which correspond to the number of wavelengths in the measured range (400–1000 nm, at approximately 1 nm intervals), not the number of independent biological samples. However, the resulting spectral curves exhibited unintended irregular fluctuations due to the interaction of multiple factors, such as the inherent noise of the camera sensor, the inhomogeneity of sample placement, and the uncertainty of the acquisition environment. To enhance the stability of the spectral data, we refined the dataset to 943 points. Through this optimization, the raw spectral data of Tencha were successfully stabilized, with the average spectral results corresponding to each processing step, detailed in Figure 3A.

Under various processing steps for Tencha, although reflectance varies across the spectral range of all samples collected, they generally exhibit similar curve trends. Particularly in the ranges of 500–600 nm and 750–950 nm, the spectral curves exhibit distinct dual peaks in reflectance. Previous studies indicate that tea leaves have lower absorption of green (490–560 nm) light in the visible spectrum and predominantly absorb red (620–780 nm) light, closely correlated with pigment content in tea leaves [19,38].This experiment observed that, as the Tencha processing steps progressed, the color of the tea leaves gradually deepened (Figure 1), further confirming this conclusion. The spectrum of visible near-infrared spectroscopy corresponds to functional groups in TFAA, such as -NH_2_, -COOH, and -OH [39]. Particularly around 947 nm, the bending of O-H bonds in theanine monomers generates strong vibrations in the first overtone region, thereby enhancing the tea’s light absorption capacity [40]. Additionally, near the 1000 nm wavelength (approaching the upper limit of the measured range), stretching of N-H bonds in TFAA molecules creates a prominent reflection region in the spectrum [41].

In Figure 3B, violin plots combined with box plots show the TFAA content across the three different processing steps: FLS, SF, and HD. The shapes represent the distribution of the data, with the inner box plot showing the median (central line) and interquartile range (IQR). Asterisks (*) indicate statistically significant differences between groups (*p* < 0.05) based on one-way ANOVA followed by Tukey’s HSD test. It reveals that the median TFAA content are similar across these steps, and the range of content is also comparable. The TFAA content is primarily concentrated around a level of approximately 4 across different processing steps, but statistical analysis did not reveal significant differences between the samples across the different processing steps. Only slight significant differences were observed between the first and third processes compared to the second step. This result may be due to the overall low content of TFAA in the Tencha.

### 3.2. Spectral Pretreating of Collected Samples

**Figure 4:**
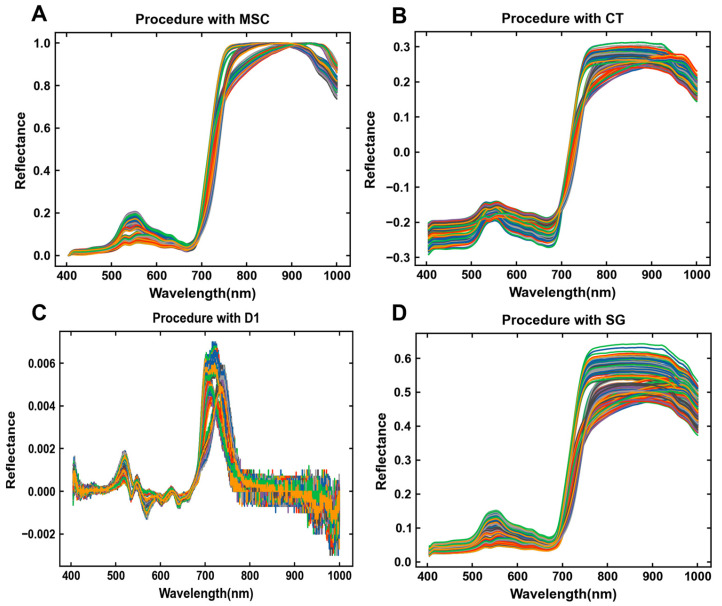
Four pretreated spectrograms for all acquisitions: (**A**) spectra after MSC; (**B**) spectra after CT; (**C**) spectra after D1; (**D**) spectra after SG.

Figure 4 shows the original spectra of all Tencha samples collected by the spectral acquisition experimental platform, as well as four different pretreated spectra. It is evident from the spectral characteristics that the pretreated spectra (Figure 4) significantly reduce baseline deviations compared to the original mean spectrum (Figure 3A).

Specifically, MSC pretreating (Figure 4A) and CT pretreating (Figure 4B) effectively remove noise, promote convergence of spectral lines, and enhance the clarity of certain spectral features. D1 pretreating (Figure 4C) notably alters the spectral shape, aiding in the separation of overlapping peaks and supporting subsequent modeling. However, after SG smoothing processing (Figure 4D), the spectral lines show minimal change compared to the original data.

The results of PLS models using different pretreating methods are shown in Table 1. From the results of the PLS models in Table 1, it is observed that, for the FLS model, the optimal predictive model is D1-PLSR, with corresponding $R_{P}^{2}$ of 0.7923 and RPD of 2.1942. For the ST model, the PLSR model after CT pretreating performs best, with $R_{P}^{2}$ of 0.7821 and RPD of 2.1423 on the prediction set. Regarding the HD model, the optimal predictive model is MSC-PLSR, with $R_{P}^{2}$ of 0.7674 and RPD of 2.0738 on the prediction set.

By comparing the predictive models of different process types in Table 1, it is found that the differences in predictive results among the three detailed models are not significant, but the best model for the FLS process performs best –with the highest $R_{P}^{2}$ and RPD values. In the CP model of the entire process flow, the CT–PLSR model exhibits the best performance, with $R_{P}^{2}$ of 0.8078 and RPD of 2.2813. However, compared with the detailed models of each process, it does not significantly improve predictive capability, indicating that the predictive performance is close to the level of detailed models. 

According to the LS–SVR model results using different pretreating methods (Table 2), the D1–LSSVR model demonstrates good predictive performance in both the FLS and other process models, with high R^2^ and RPD values, and low RMSE. For the ST model, the MSC–LSSVR model is considered the best predictive model, with $R_{P}^{2}$ = 0.7258 and RPD = 1.9097 on the prediction set. In the CT-pretreated HD process model, the LS–SVR algorithm achieves $R_{P}^{2}$ = 0.7240 and RPD = 1.9036 in predicting TFAA content of Tencha.

Table 1 and Table 2 summarize the results of PLS and LS-SVR models using different pretreating methods. The results indicate that the spectral modeling after pretreating is effective, reflected in higher *R*^2^ values and lower RMSE values on both the calibration and prediction sets. Overall, PLS performs better than LS–SVR in modeling.

Combining the different process model prediction results from Table 1 and Table 2, it is found that the detailed model prediction performance of the FLS process is superior to that of the other two processes. This may be because the FLS process, as the first step in the processing sequence, is less affected by cumulative external influences, such as operational variability, during subsequent steaming or drying stages, environmental factors (e.g., temperature or humidity fluctuations), or mechanical stresses. Consequently, the internal quality variations of the samples at this stage have a relatively direct and significant impact on the model results.

### 3.3. Selection of Characteristic Bands

#### 3.3.1. Characteristic Band Selection by CARS

**Figure 5:**
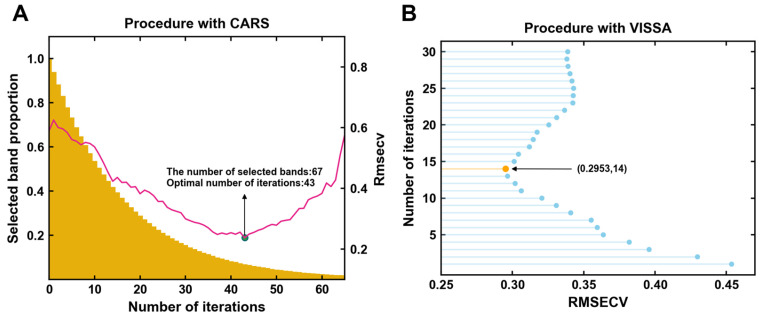
Variation of each parameter with increasing number of iterations in the CARS characteristic selection method under the best pretreated global model (**A**); Variation of the number of iterations for RMSECV during VISSA characteristic selection method under the best pretreated global model (**B**).

In the PLSR–CP model, when using the CARS characteristic selection method, the proportion of selected wavelengths gradually decreases relative to the total number of wavelengths, indicating effective reduction in data dimensions and redundancy in the original spectrum (Figure 5A). Unlike VIP scores derived from the PLSR model, which rank variables based on importance but do not eliminate redundancy, CARS focuses on selecting a sparse subset of key wavelengths that optimize model performance [42]. This makes it particularly suitable for simplifying high-dimensional spectral datasets, aligning with the goals of this study.

**Figure 6:**
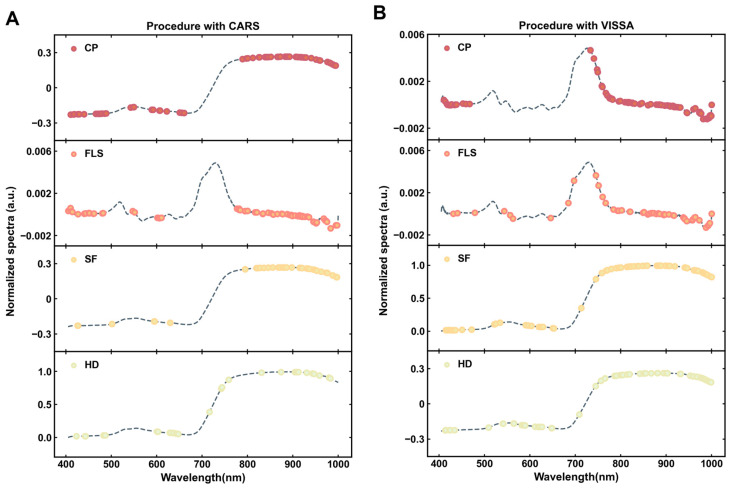
Plot of band positions for CARS characteristic selection in models after optimal pretreatment (**A**); plot of band positions for VISSA characteristic selection in models after optimal pretreatment (**B**).

The CARS algorithm evaluates the performance of different combinations of characteristic bands using RMSECV to select the optimal characteristic bands. In this algorithm, the Monte Carlo sampling number (N) is set to 65, and RMSECV in 5-fold cross-validation shows a trend of first decreasing and then increasing with increasing N. At the 43rd iteration, RMSECV reaches a minimum value of 0.2369. Combined with the selection of the optimal CT pretreating method in Table 1, the CARS algorithm illustrates in Figure 6A the positions of the selected characteristic bands on the average spectrum. At this point, the optimal number of characteristic bands is 67, accounting for 23.7% of the total number of bands.

In the PLSR-detailed models for the three processes of FLS, SF, and HD that have been seen in this study (Table 1), the optimal pretreatment is D1, CT, and MSC, respectively. The corresponding characteristic bands are also screened by Figure 6A using the CARS algorithm for the three detailed models, and a total of 67, 41, and 32 characteristic variables, which account for 7.1%, 4.3%, and 3.3% of the total wavelengths, respectively, have been obtained. The spectral matrix dimension is effectively reduced.

#### 3.3.2. Characteristic Band Selection by VISSA

Under the global model containing all the process samples, Figure 5B reflects the variation of the root mean square error cross-validation value (RMSECV) during each iteration run of the VISSA characteristic selection algorithm after optimal pretreating. Before the number of running iterations is 14, the RMSECV shows an overall decreasing trend, which is due to the fact that many wavelengths that are not related to the moieties contained in the TFAA are removed during the iteration process, but thereafter the RMSECV gradually increases with the further increase in the number of iterations, which may be due to the addition of some irrelevant wavelengths or the deletion of some relevant wavelengths during the iteration process.

Figure 6B shows the final number of bands and location information for the four groups of optimal models obtained after pretreating and screened at the end of the VISSA algorithm. VISSA aims to select representative bands that capture critical variations in the spectral data. However, as seen in Figure 6B, some redundancy may persist due to overlapping contributions from similar molecular vibrations. While VISSA effectively reduces the dimensionality of the spectral data and selects relevant features, it may include redundant bands associated with the same molecular contributions. This highlights the trade-off between dimensionality reduction and maintaining spectral information richness, which is a common challenge in feature selection for hyperspectral data. Meanwhile, the CP model containing samples from all the processes obtained relatively fewer spectral bands that were selected, which also implies that there is a stronger correlation between these bands and the TFAA in Tencha.

### 3.4. Results of Regression Model Corresponding to Different Characteristic Selection Methods

Table 3 illustrates that the application of two characteristic band selection methods reduces the dimensionality of the spectral matrix in the sample data, thereby significantly accelerating the modeling process. Following the characteristic band screening using the CARS algorithm, the predictive performance of most models was significantly enhanced, with the CP global model of CT-CARS-PLSR exhibiting the best performance, as indicated by the predictive indicators: $R_{C}^{2}$ = 0.9885, $R_{P}^{2}$ = 0.9566, RMSEC = 0.0956, RMSEP = 0.1749, and RPD = 4.8021. This suggests that the CARS method effectively eliminates irrelevant interfering information from the spectral data that does not pertain to TFAA.

In Table 4, the modeling results after characteristic variable selection using the VISSA algorithm demonstrate good predictive performance under various processing conditions, effectively eliminating collinearity between different wavelengths and simplifying the model. Among them, under the CP global process, the D1-VISSA-LSSVR model shows the best predictive performance, with specific indicators of $R_{C}^{2}$ = 0.9856, $R_{P}^{2}$ = 0.1150, RMSEC = 0.9304, RMSEP = 0.1841, and RPD = 3.7924. However, the improvement in predictive performance of certain individual models (such as the two optimal models under the HD process) is limited, further reflecting that the VISSA algorithm, due to its iterative nature, can maintain a high prediction accuracy while selecting relatively fewer characteristic bands, thereby avoiding overfitting issues. However, this may also lead to the inability to capture some target spectral information.

**Figure 7:**
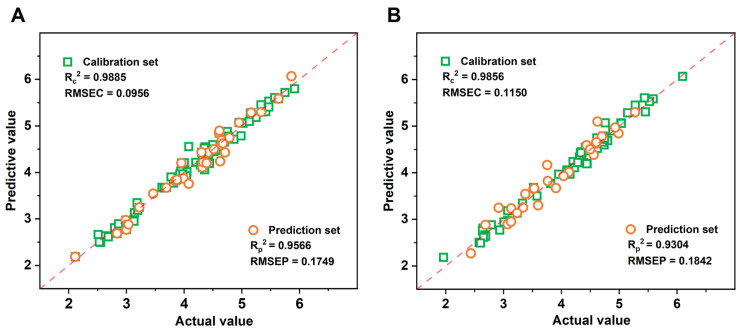
Scatterplot of the best model under different modelling approaches: (**A**) CT–CARS–PLSR; (**B**) D1–VISSA–LSSVR.

Compared with models established under the same conditions, the model performance after CARS characteristic variable selection is slightly better, especially suitable for optimizing the prediction model of TFAA content in Tencha. Figure 7 shows the prediction results under different modeling methods, with CT–CARS–PLSR and D1–VISSA–LSSVR being the top two models. The dashed line in the figure represents the linear regression relationship between model predicted results and actual values. In Figure 7A, the data points are narrowly distributed around the regression line. Combined with the higher *R*^2^ and RPD shown in Table 4, as well as the lower RMSE, it indicates that linear PLSR has a superior predictive level compared to nonlinear LS–SVR. Therefore, CT–CARS–PLSR can be considered the most suitable model for predicting TFAA content in Tencha. Although the FLS process plays a key role due to its direct reflection of internal quality, the superior performance of the global model demonstrates that the information contributed by the subsequent processing steps (SF and HD) is also crucial. These steps, while less influential on their own, capture additional spectral variations and contribute to a more comprehensive understanding of Tencha quality when integrated into a unified model.

### 3.5. Graphical Visualization of Model Results

**Figure 8:**
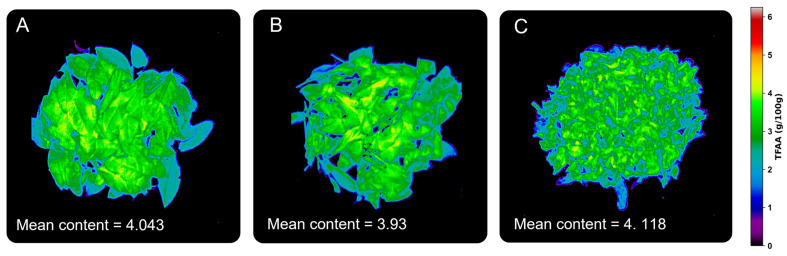
Visualization of amino acid content in milled tea from different processes based on visible NIR spectroscopy; (**A**) FLS processing; (**B**) SF processing; (**C**) HD processing.

The VNIR–HSI technique not only provides characteristic spectra of substances but also effectively reflects the chemical composition and internal structural information of materials. In this study, the selected optimal model was used to visually predict the TFAA content in Tencha samples processed under three different processing steps. Figure 8 shows the visible near-infrared spectroscopy images of the processed green tea leaves, where pseudo-color indicates the TFAA content in the leaves after different processing steps. Observations from the figure reveal that, as the processing progresses, the internal moisture of the tea samples gradually decreases, the surface color deepens progressively, and the leaf morphology changes from unfolded to curled, ultimately becoming fragmented. However, the TFAA content does not show significant differences within these three processing steps, consistent with the results of related studies [43]. Additionally, this study also highlights the critical role of visualizing TFAA content in assessing the quality of Tencha on the production and processing flow line, ensuring consistency in the final product’s quality and the desired sensory characteristics.

## 4. Conclusions

Combining visible-near infrared spectroscopy, we collected hyperspectral data of green tea samples processed using three different techniques and determined the AA content in each corresponding sample. We established and compared detailed prediction models for each processing technique as well as an overall prediction model incorporating all techniques. By employing various pretreating and characteristic wavelength selection methods, we effectively optimized the prediction accuracy of these models and successfully visualized the predicted TFAA content.

Furthermore, slight differences were observed among the detailed prediction models established for each processing technique, with the FLS processing showing particularly prominent predictive performance. This reflects that the initial processing stages can reduce internal interference factors in tea leaf samples, thereby benefiting model performance.

Although the experimental results demonstrate that VNIR–HSI technology can rapidly and accurately detect TFAA content in tea samples, providing crucial technical support for upgrading tea processing quality, it is noteworthy that our study used a limited number of samples and only quantitatively analyzed TFAA in a single variety of Tencha. Therefore, comparative analysis across different varieties is lacking. Additionally, individual amino acids such as glutamic acid and theanine are closely related to the flavor and quality of tea, suggesting that future research directions should include more refined spectral analysis of individual amino acids.

## Figures and Tables

**Table 1:** PLSR model for prediction of TFAA content of Tencha under different pretreatment methods.

Prediction Model	Pretreatment	Calibration Set		Prediction Set		RPD
		$R_{C}^{2}$	RMSEC	$R_{P}^{2}$	RMSEP	
CP	MSC	0.7403	0.5453	0.7572	0.6903	2.0297
	**CT**	**0.8515**	**0.4376**	**0.8078**	**0.5042**	**2.2813**
	D1	0.9691	0.1880	0.7843	0.6506	2.1534
	SG	0.9188	0.3048	0.7634	0.6815	2.0558
FLS	MSC	0.8899	0.3549	0.7603	0.6859	2.0428
	CT	0.7453	0.5401	0.7779	0.6602	2.1221
	**D1**	**0.9739**	**0.1834**	**0.7923**	**0.5242**	**2.1942**
	SG	0.8488	0.4160	0.7463	0.7056	1.9855
ST	MSC	0.9007	0.3372	0.7315	0.7260	1.9299
	**CT**	**0.8816**	**0.3907**	**0.7821**	**0.5369**	**2.1423**
	D1	0.9262	0.2907	0.7498	0.7006	1.9999
	SG	0.8878	0.3804	0.6982	0.6319	1.8204
HD	**MSC**	**0.8687**	**0.4114**	**0.7674**	**0.5547**	**2.0738**
	CT	0.9007	0.3372	0.7315	0.7260	1.9299
	D1	0.9067	0.3268	0.7133	0.7501	1.8678
	SG	0.9083	0.3239	0.7462	0.7057	1.9855

**Table 2:** LS–SVR model for prediction of TFAA content of Tencha under different pretreatment methods.

Prediction Model	Pretreatment	Calibration Set		Prediction Set		RPD
		$R_{C}^{2}$	RMSEC	$R_{P}^{2}$	RMSEP	
CP	MSC	0.8042	0.4735	0.7700	0.6719	2.0853
	CT	0.8444	0.4409	0.7786	0.5631	2.1252
	**D1**	**0.8559**	**0.4244**	**0.7882**	**0.5507**	**2.1733**
	SG	0.9576	0.1901	0.7036	0.4530	1.8368
FLS	MSC	0.8947	0.3626	0.7187	0.6347	1.8856
	CT	0.8367	0.4588	0.7240	0.6043	1.9036
	**D1**	**0.9419**	**0.2692**	**0.7685**	**0.5757**	**2.0785**
	SG	0.8142	0.4894	0.6899	0.6405	1.7958
ST	**MSC**	**0.9759**	**0.1312**	**0.7258**	**0.5309**	**1.9097**
	CT	0.7880	0.4927	0.7008	0.7663	1.8282
	D1	0.9067	0.3268	0.7133	0.7501	1.8678
	SG	0.9027	0.3337	0.6721	0.8022	1.7465
HD	MSC	0.8878	0.3804	0.6982	0.6319	1.8204
	**CT**	**0.8367**	**0.4588**	**0.7240**	**0.6043**	**1.9036**
	D1	0.8809	0.3691	0.7164	0.7461	1.8778
	SG	0.7038	0.5824	0.7116	0.7523	1.8623

**Table 3:** Calibration and prediction results of different modelling methods after CARS characteristic selection.

Models	The Type of Process	Calibration Set		Prediction Set		RPD
		$R_{C}^{2}$	RMSEC	$R_{P}^{2}$	RMSEP	
PLS	**CP**	**0.9885**	**0.0956**	**0.9566**	**0.1749**	**4.8021**
	FLS	0.9849	0.1179	0.9278	0.1875	3.7236
	SF	0.9898	0.1334	0.8837	0.4986	2.9326
	HD	0.9792	0.1386	0.8982	0.2228	3.1344
LSSVM	**CP**	**0.9855**	**0.1112**	**0.8571**	**0.3145**	**2.6456**
	FLS	0.9581	0.1871	0.8512	0.3373	2.5926
	SF	0.9699	0.1584	0.8343	0.3559	2.4571
	HD	0.9831	0.1248	0.8124	0.3037	2.3046

**Table 4:** Calibration and prediction results of different modelling methods after VISSA characteristic selection.

Models	The Type of Process	Calibration Set		Prediction Set		RPD
		$R_{C}^{2}$	RMSEC	$R_{P}^{2}$	RMSEP	
PLS	**CP**	**0.9343**	**0.2355**	**0.8302**	**0.5772**	**2.4273**
	FLS	0.9264	0.2480	0.8257	0.3651	2.3953
	SF	0.9829	0.1195	0.8112	0.3801	2.3012
	HD	0.9706	0.1566	0.7874	0.4032	2.1690
LSSVM	**CP**	**0.9856**	**0.1150**	**0.9304**	**0.1841**	**3.7924**
	FLS	0.9797	0.1316	0.8873	0.2793	2.9793
	SF	0.9675	0.1667	0.8412	0.3316	2.5096
	HD	0.9873	0.1042	0.8366	0.3364	2.4739

## Data Availability

The data presented in this study are available on request from the corresponding author.
